# Precise Phase Measurement for Fringe Reflection Technique through Optimized Camera Response

**DOI:** 10.3390/s23239299

**Published:** 2023-11-21

**Authors:** Fendan Hu, Wenqi Zhu, Wei Huang, Jinshan Xu

**Affiliations:** College of Computer Science & Technology, Zhejiang University of Technology, Hangzhou 310023, China; 1111912004@zjut.edu.cn (F.H.); aizhuwenqi@icloud.com (W.Z.); huangwei@zjut.edu.cn (W.H.)

**Keywords:** fringe reflection technique, precision improvement, camera response function

## Abstract

The Fringe Reflection is a robust and non-contact technique for optical measurement and specular surface characterization. The periodic alternation between dark and light cycles of the fringe patterns encodes the geometric information and provides a non-contact method of spatial measurement through phase extraction. Precisely expressing the positions of the points of the fringe pattern is a fundamental requirement for an accurate fringe reflection measurement. However, the nonlinear processes, both in generating the fringe pattern on a screen and capturing it using pixel values, cause inevitable errors in the phase measurement and eventually reduce the system’s precision. Aiming at reducing these nonlinear errors, we focus on constructing a new quantity from the pixel values of the photos of the fringe patterns that could linearly respond to the ideal fringe pattern. To this end, we hypothesize that the process of displaying the fringe pattern on a screen using a control function is similar to the process of capturing the pattern and converting the illuminating information into pixel values, which can be described using the camera’s response function. This similarity allows us to build a scaled energy quantity that could have a better linear relation with the control function. We optimize the extracted camera response function using an objective to increase the precision and reduce the quoted error. Experiments designed to determine the positions of points along the quartile lines verify the effectiveness of the proposed method in improving fringe reflection measurement precision.

## 1. Introduction

The Fringe Reflection or the deflectometry is a robust technique that provides a non-contact and accurate method for optical measurements and specular surface characterization [1,2]. This technique is derived from the interferometry theory, as a grating pattern shows distortion in the mirror image through an imperfect specular surface [3]. Consequently, comparing the deformed mirror image with a standard reference pattern, the imperfection of the specular surface can be inferred [4,5]. Thanks to its high-speed inspection and measurement precision, this technique is applicable in many different fields, such as in window shields and car bodies in the automobile industry [6], solar collectors [7,8], etc.

With the increased demand for clean and renewable energy, solar thermal power has been receiving more and more attention, due to its high sun-to-electricity conversion ratio. The solar collector, which is composed of a set of reflective mirrors of a particular shape, is one of the key components in such a system. Fabricating a high-quality solar collector (mirror facets) is not only a requirement of high efficiency but a requirement of safety [9]. Measurement techniques that are capable of characterizing the quality of the solar collector are required in the production line, and are continuously receiving research attention [10]. The fringe reflection is an ideal tool for measuring the optical properties, such as the local surface slopes, focal length, twist errors, etc. [11]. The adaptation and improvement of this technique have been hot research topics in solar thermal power development. The pioneer application can be dated back to 2003, when Fontani et al. [12] introduced the reflecting grating moire method in measuring the curvature of mirrors used in a heliostat plant, and achieved an accuracy of 0.1 mrad for surface gradients [13]. The milestone achievement was made by Andraka and co-authors in the Sadia National Laboratories, who developed the Sandia Optical Fringe Analysis Slope Tool (SOFAST) [14]. This fringe-reflection-based tool not only enables the fast characterization of mirror facets’ shape and rotations in almost real-time [15], but also the development of the Alignment Implementation for Manufacturing using Fringe Analysis Slope Technique (AIMFAST) [16].

No matter the field in which this technique is applied, the basic setup is the same, i.e., a standard fringe pattern is displayed on a screen and a camera takes photos of the distorted fringe pattern through the reflection of the mirror to be tested [17]. In order to reconstruct a full map of the specular surface, it is essential to calculate the normal vector of each reflective point. Typically, this procedure is accomplished by applying the ray-tracing algorithms to the source point on the screen, its corresponding image point in the photo and reflecting point on the mirror, as long as the positions of these three points are expressed in the same coordinate system [18]. Thanks to the algorithm that precisely retrieves the transforming matrix between the world and camera coordinate systems [19], the expression of positions in different coordinate systems can be unified. As a result, automatically determining the position of a source point on the screen in its local coordinate system is a basic requirement of precise measurement.

Phase shifting is the frequently used method in determining the position of a point in the screen coordinates. To proceed, a series of fringe patterns with a fixed phase difference in one dimension are generated and captured through the reflection of the test mirror. With the pixel values of a point recorded in different photos, its position is then calculated by applying the phase shifting algorithm, which transforms the pixel values into an absolute phase of that particular point [10]. Further combining the periodicity of the fringe pattern, its position is then obtained. As the phase shift algorithm is derived from the assumption of an ideal pattern shape [20], its accuracy is sensitive to any deformation to the fringe pattern caused by the nonlinear response of the camera in capturing the projected pattern. For example, image saturation can severely affect measurement precision [21].

Due to the collective awareness of the effect of a camera’s nonlinear response on the measurement precision, great research efforts have been dedicated to improving the phase-shifting algorithms. For example, Lei et al. [22] proposed a new phase extraction algorithm based on the Gram–Schmidt orthonormalization and least square ellipse fitting. Zhai et al. [23] developed a general phase extraction algorithm by utilizing the Lissajous figures and ellipse fitting. Wang et al. [24] proposed the triple N-step phase shift algorithm to compensate for the errors in fringe projection. Han et al. introduced a two-step calibration procedure in the measuring phase in the deflectometry system [25]. Tounsi et al. proposed a digital four-step phase-shifting technique. It enables a precise phase measurement using only one fringe pattern, as the fringe patterns of different phase shifts are generated from the first-, second-, and third-order Riesz transform components of the original one [26]. In 2022, Chang et al. [27] proposed the adaptive phase shift and integrated it into the multi-surface interferometric algorithm.

Notice that the saturation of shooting fringes due to ambient light, or the characteristics of the object to be measured, are the most severe nonlinear effects; researchers have tried to introduce different modulations to alleviate the camera’s nonlinearity in responding to the projected fringe pattern, especially to avoid gray saturation. Chang et al. proposed the use of an infrared sinusoidal pattern and infrared camera to avoid the interference of ambient light [28]. Waddington and Kofman [29] adaptively adjusted the maximum input gray level (MIGL) of the projected fringe pattern and the composite images captured at different exposure intensities to avoid image saturation. After this work, an adaptive fringe pattern was designed [30], which generates patterns of appropriate intensity in regions according to their local optical properties. Apparently, this requires complex pre-calibration. As with the method proposed by Lin et al. [31], a set of uniform gray pattern sequences are required in order to determine the best projection gray level. Recently, Wang et al. introduced a linear interpolation to tackle pixel saturation [21].

The existing works mainly focus on the nonlinearity in the process of converting an illumination pattern into pixel values, with a preset assumption that the projected pattern keeps its ideal form. In fact, the nonlinearity exists not only in the process of converting the scene illumination into pixel values, but also in generating the fringe patterns on the screen. When an ideal fringe pattern generated by some control function (computer program) is displayed on a screen, deformation is also inevitable. The fringe pattern on the screen itself might not be of an ideal shape in terms of gray value or brightness. This distorted fringe pattern would severely reduce the precision of the measurement system, and even the projected pattern could be captured accurately. Consequently, establishing a linear relation between the pattern illumination and the corresponding pixel values might not guarantee a precise phase measurement. This paper aims to reduce the errors caused by a set of nonlinear processes involved in the fringe-reflection-based measurement. The key idea is to establish a quantity that responds to an ideal fringe pattern in a linear way. In the following, we first present the principle of applying the fringe reflection in concentrative mirror facets’ qualification and its limitations. We detail the procedures of establishing the suitable quantity in Section 3. The experimental results immediately follow in order to validate the effectiveness of the proposed method in Section 4. We end the paper with a short conclusion on the main findings of the paper and a discussion of the limitations.

## 2. Fringe Reflection Principle and Application Limitations

**Figure 1:**
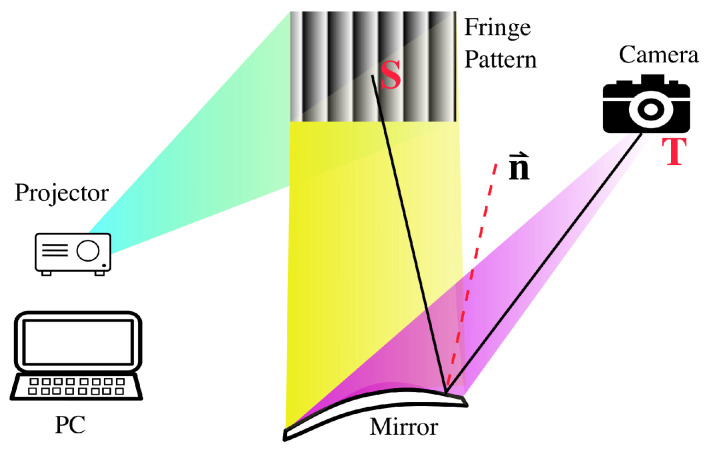
Reflective mirror surface quality examination system, i.e., a typical application of the fringe pattern technique. A computer program generates the sinusoidal fringe pattern on a monitor screen. Any point S on the screen will be captured by the camera through the reflection of the mirror to be measured. Pairing up the point S and its image point T determines the normal vector **$\vec{n}$** and, finally, the mirror quality [18].

Fringe projection is an ideal technique for large area measurements, providing high accuracy and repeatability with low system noise. When being applied to measure large reflective mirror surfaces [18], a fringe projection system typically consists of a projector and a camera. As shown in Figure 1, the projector projects a fringe pattern (a sinusoid pattern) generated using a computer program onto a large screen, which can be captured by the camera through the reflection of the mirror to be measured. By pairing a target point **T** in the image from the camera with its source point **S** on the screen, a unit normal vector $\vec{n}$ characterizing the optical property of the mirror surface can be fully determined [18]. Extracting the spatial information of points on the screen automatically and precisely is a fundamental requirement of the subsequent measurement.

### 2.1. Four-Step Phase-Shifting Algorithm

The periodicity of the fringe pattern serves as a ruler in determining the coordinates of any point on the screen. However, due to the interference of background illumination, a direct measure of the intensity of the projected pattern cannot provide precise spatial information. Phase-shifting is such a technique that eliminates background interference. In the past decades, scholars have developed a variety of phase-shifting algorithms, among which the four-step phase-shifting algorithm is the most frequently used. To proceed, a set of sinusoidal patterns of the same form as Equation (1) are projected onto the screen:

(1) $$G_{i}\left(x,y\right)=G_{0}\cos\left(\phi \left(x,y\right)+\delta_{i}\right)$$

where $\delta_{i}$ ($=0,\frac{\pi }{2},\pi ,\frac{3\pi }{2}$) is the phase shift between the two sinusoidal patterns, *x* and *y* are the coordinates of the screen, and $G_{0}$ is the amplitude of fringe modulation. By recording the intensities $G_{i}\left(x,y\right)$ at point (x,y) under the illumination of the fringe patterns of the phase shift $\delta_{i}$, we can determine the phase ϕ at (x,y) as

(2) $$\tan\phi \left(x,y\right)=\frac{G_{4}-G_{2}}{G_{1}-G_{3}}$$

The advantage of using phase-shifting is clearly manifested in Equation (2), where the interference of background illumination is eliminated by taking the difference between the measured pattern intensities $G_{i}$. Further combining the periodicity of the fringe pattern in space, the coordinates of a point can be determined as follows:

(3) $$x=\frac{\lambda }{2\pi }\phi =\frac{\lambda }{2\pi }\cdot \tan^{-1}\left(\frac{G_{4}-G_{2}}{G_{1}-G_{3}}\right)$$

where λ represents the wavelength of the projected sinusoidal patterns.

### 2.2. Error Analysis in Practical Use

**Figure 2:**
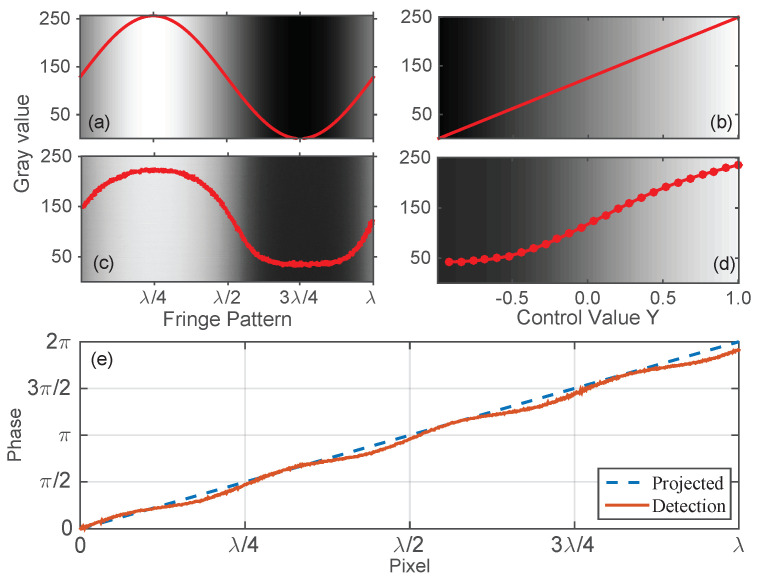
Illustration of the effect of the camera’s nonlinear response in phase measurement. (**a**) Ideal fringe pattern generated using a control function Y∝cos(). (**b**) The expected response to the control value *Y*. (**c**) A deformed sinusoidal fringe pattern captured using a digital camera. (**d**) Camera’s real response to the control value *Y*, which is clearly not linear. (**e**) The effect of deformed fringe pattern in phase measurement.

Since Equation (2) is derived from an ideal sinusoidal pattern, the precision of the extracted spatial information severely depends on the quality of the sinusoidal fringes captured by the camera. When an ideal sinusoidal pattern is projected onto the screen (Figure 2a), we would expect that its intensity could be linearly captured by the camera (panel (b)). However, as the camera responds to sense illumination in a nonlinear way [32], the captured pattern distorts, i.e., the fringe pattern is no longer of a sinusoidal form (panel (c)). Using this distorted fringe pattern in Equation (2) brings unexpected errors to phase measurement and, finally, the extracted spatial information, especially when the illumination is relatively low and high due to dark noise and saturation, respectively (see Figure 2e).

Ideally, we project a fringe pattern of intensity range I∈[a,b] generated using a computer program onto the screen, and expect that the camera linearly maps this pattern into pixel values *G*, i.e., G∝I (Figure 2b). However, in reality, the generated pixel value *G* in responding to illumination intensity $I_{i}$ would be of the following form:

(4) $$G_{i}=cI_{i}+N\left(I_{i}\right)$$

where $N(I_{i})$ represents the high-order responses to illumination $I_{i}$, and *c* is a constant representing the linear part which can be set to 1 during analysis without any loss of generality. When applying these $G_{i}$ in measuring the phase, Equation (2) would become

(5) $$\phi =\arctan\left(\frac{\left(I_{4}+N\left(I_{4}\right)\right)-\left(I_{2}+N\left(I_{2}\right)\right)}{\left(I_{1}+N\left(I_{1}\right)\right)-\left(I_{3}+N\left(I_{3}\right)\right)}\right)$$

As can clearly be seen from Equation (5), the measurement in phase is sensitive to the nonlinearity of the camera response. However, this nonlinearity comes from the fundamental physical rules of the imaging devices [32]. Developing algorithms that help in reducing the nonlinearity is of essential importance in increasing the precision of the fringe-pattern-based measurement [18].

## 3. Camera-Response-Function-Based Fringe Measurement

As mentioned above, the errors of the conventional fringe pattern measurement are a direct consequence of the imperfect sinusoidal pattern captured using the camera. Unfortunately, there is no explicit knowledge on how and to what extend the pattern is deformed from the ideal shape, as there are multiple nonlinear processes involved, i.e., the projector generates a pattern on the screen according to a control function Y∝cos(·), then the camera represents the pattern with pixel values. All these nonlinear responses integrally contribute to the deformation, as seen in Figure 2. As a result, extracting quantities that map the intended sinusoidal pattern linearly is essential in increasing measurement precision.

### 3.1. Fringe Pattern Generation and Acquisition

Recall the procedure of fringe measurement, i.e., a pattern is typically generated using some computer programs according to the data of the form Y∝cos(·). Using this information *Y*, the projector casts a pattern of irradiance *E* on the screen. The image sensor of the camera responds to the irradiance *E* by producing an electronic signal and is further digitized into a pixel value *G*. Although the pixel value *G* responds to *Y* monotonically, thisis not in a linear way (see Figure 2d). During the whole process, the pixel value *G* is the only quantity we can obtain. Consequently, constructing a new quantity *y* that is proportional to *Y* from the pixel value *G* is a key step in fringe measurement.

However, the sense irradiation *E* is not the only factor that determines the resulting pixel value *G*. The exposure time δt plays the same role. The longer the exposure time, the greater the energy that is absorbed. So, the camera response curve shows a monotonical increase. This behavior is described by the response function $G=F\left(E\cdot \delta t\right)$, where F(·) describes how the camera converts the absorbed energy E·δt into a pixel value *G*. Since the exposure time δt is independent of the fringe pattern, it is reasonable to exclude the exposure time from the measured quantity in order to better represent the fringe pattern. That is to say, we need to reconstruct an inverse function $F^{-1}$ that extracts the illumination information *E* from the pixel value *G* as

(6) $$E=F^{-1}\left(G\right)$$

Equation (6) is known as the camera’s responding function. In 1997, a method was proposed by Debevec and Malik [33] for recovering this function, i.e., obtaining the illumination information (irradiation *E*) using an explicit function lnE=g(G), with g(·) a polynomial function [18].

Unfortunately, the illumination information *E* alone would not give us satisfactory results, as the key requirement of a precise fringe technique is the linear response to the controlled fringe pattern. Diving deeper in the process of pattern generation and acquisition, generating the fringe pattern using a control function *Y* could be seen as an inverse process of converting irradiation *E* into pixel value *G*. Consequently, a linear relationship between *Y* and lnE is expected, and, consequently, a more precise measurement.

### 3.2. Linear Response Establishment

#### Camera Response Function

**Figure 3:**
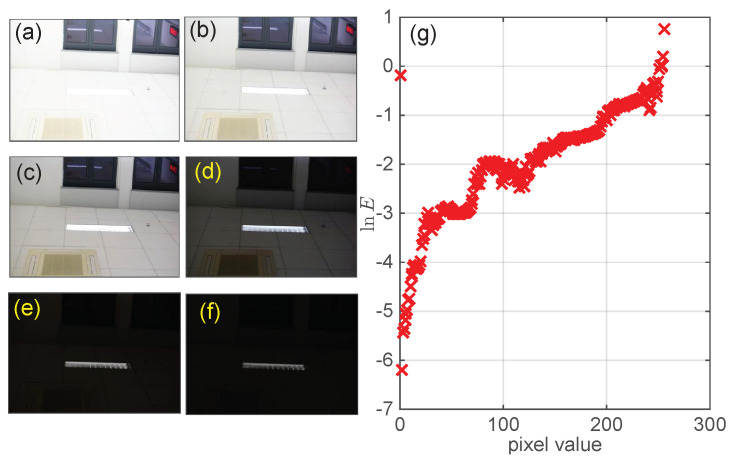
(**a**–**f**) Photos of one scene taken with different exposure times: (**a**) 1/2, (**b**) 1/10, (**c**) 1/30, (**d**) 1/60, (**e**) 1/80, and (**f**) 1/100, respectively. (**g**) The camera’s response function extracted from Equation (9) using these images.

The analysis in the previous sub-section implies the importance of illumination information *E* in establishing a linear relation between an ideal sinusoidal pattern and the only quantity *G* that can be measured. The method developed by Debevec and Malik is frequently used to extract this relation [33], and the precision of this method has been widely validated [34,35,36]. To proceed, a set of *M* photos of a scene are taken with different exposure times $\delta t_{j}$ over a short period, as shown in Figure 3. We then express the camera’s response function F that converts the total energy $E_{i}\delta t_{j}$ received at point *i* into pixel values $G_{i}^{j}$ in the *j*th photo as follows:

(7) $$F^{-1}\left(G_{i}^{j}\right)=E_{i}\delta t_{j}$$

**Figure 4:**
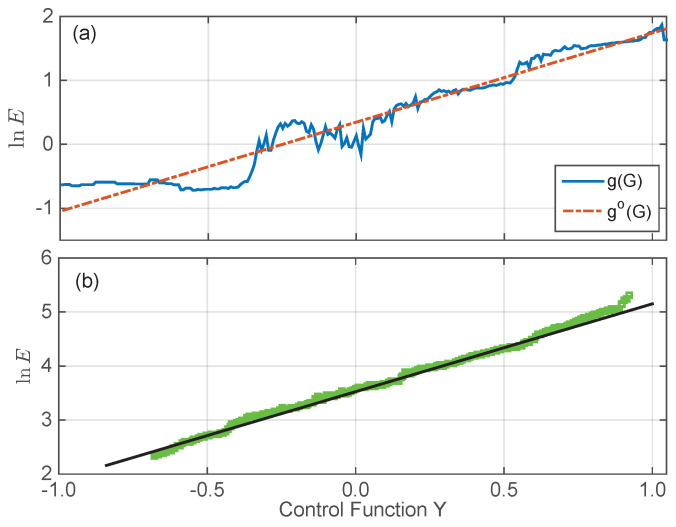
(**a**) Determination of the weight function *w* by calculating the difference between the previously obtained lnE=g(G) with its best fit $g^{o}\left(G\right)$ to the control value *Y*. (**b**) The response of lnE extracted from Equations (10)–(12) to control value *Y*. Green dots are obtained lnE using response function constructed from Equations (10)–(12).

The positive definiteness of pixel values and irradiation strength allow us to take logarithm operations on both sides of Equation (7), and to turn them into

(8) $$\ln F^{-1}\left(G_{i}^{j}\right)=g\left(G_{i}^{j}\right)=\ln E_{i}+\ln\delta t_{j}$$

where $g(G_{i}^{j})$ is the explicit form of $\ln F^{-1}\left(G_{i}^{j}\right)$, which can be approximated by a *K*th polynomial of *G* as $g\left(G\right)=\sum_{k=0}^{K}a_{k}G^{k}$. Determining the coefficients $a_{k}$ is then transformed into minimizing a quadratic objective function [33]

(9) $$O=\sum_{i=1}^{N}\sum_{j=1}^{M}{\left[g\left(G_{i}^{j}\right)-\ln E_{i}-\ln\delta t_{j}\right]}^{2}+\gamma \sum_{G=G_{min}+1}^{G_{max}-1}{\left[g^{\prime \prime }\left(G\right)\right]}^{2}$$

where $G_{min}$ and $G_{max}$ are the smallest and largest pixel values among the total *N* pixels in all figures, and a regularization term with scaler γ (= 1.0) weights the smoothness relative to the data fitting. Applying the singular value decomposition method to Equation (9), we extract g(G) and show it in Figure 3g. Apparently, there is no simple relation between the captured pixel values and their corresponding illumination energy *E*. With the extracted relationship G=f(Y) between the control value *Y* and the captured pixel values *G* shown in Figure 2d, we obtain the relation between *Y* and lnE (see the solid curve in Figure 4a). However, when we extrapolate these two quantities with a linear relation $\ln E=g^{o}\left(Y\right)$ (see the dashed line in Figure 4a), large residual errors are observed.

Since finding a quantity that responds linearly to the control value *Y* used in generating the ideal sinusoidal pattern is essential to the precision of any fringe-based measurement, we re-design the objective function by introducing a weight w(G) to the quadratic term in Equation (9), with the purpose of retrieving the camera’s response function g(·). As the linearity is directly linked to precision, we want the weight w(G) to be helpful in minimizing the residual errors ε between g(G) and its linear extrapolation $g^{o}\left(G\right)$ (see the dash-dotted line in Figure 4a). Combining all the above-mentioned requirements together, we come up with a modified quadratic objective function:

(10) $$O=\sum_{i=1}^{N}\sum_{j=1}^{M}w\left(G_{i}^{j}\right){\left[g\left(G_{i}^{j}\right)-\ln E_{i}-\ln\delta t_{j}+\varepsilon_{i}\right]}^{2}+\gamma \sum_{G=G_{min}+1}^{G_{max}-1}{\left[g^{\prime \prime }\left(G\right)\right]}^{2}$$

(11) $$\varepsilon_{i}=g^{1}\left(f(Y_{i})\right)-g^{0}\left(f(Y_{i})\right)$$

(12) $$w\left(G_{ij}\right)=\left\{\begin{array}{cc} 0 & \;G_{i}^{j}<G_{min}\;\&\;G_{i}^{j}>G_{max}\\ \frac{1}{1\;+\;\left|\varepsilon_{i}\right|} & \;G_{min}\leq G_{ij}\leq G_{max} \end{array}\right.$$

where the weight $w\left(G_{ij}\right)$ is introduced to guarantee a satisfactory linearity near the middle point of the control value *Y*. As expected, the optimal solution of Equations (10)–(12), i.e., g(G), shows clear linear dependency on the control value *Y*, especially when *Y* is close to zero (see Figure 4b).

**Figure 5:**
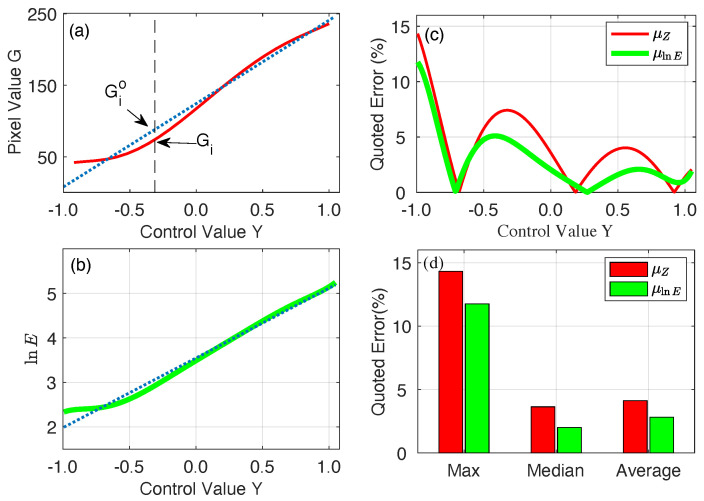
Pixel value *G* (**a**) and scaled energy lnE (**b**) representation of camera’s response to control value *Y*. (**c**) The quoted errors of these two representations and different measurement points. (**d**) Comparison of the max, mean, and median quoted errors of the two camera response representations.

Recall the procedure of applying the four-step phase-shift principle in fringe measurement, the assumption of a linear response to an ideal sinusoidal fringe pattern is a pre-requirement. That is to say, to precisely measure the phase ϕ using Equation (2), the quantities $G_{i}$ have to have a linear relation with the control value *Y*. When pixel values $G_{i}$ are used (see Figure 5a), for each control value $Y_{i}$, its corresponding $G_{i}^{o}$ on the dotted line must be used. However, due to the nonlinear response of the camera’s pixel values to the control value *Y*, it is $G_{i}$ that is used in reality. The difference between $G_{i}^{o}$ and $G_{i}$ causes inevitable errors in measurement. This is termed quoted error, which is a intrinsic property of a measurement system. In order to set a reasonable basis for comparing different systems, the quoted error is typically expressed as follows:

(13) $$\mu_{G}^{i}=\frac{∥G_{i}^{o}-G_{i}∥}{L}\times 100\%$$

where *L* is the measurement range of the system. In the case of using pixel values, *L* would be defined as $G_{max}-G_{min}$. Throughout the whole measurement range, the quoted error is different from point to point. As can be seen from Figure 5b, using the pixel values *G* in measuring the phase gives rise to high quoted errors, especially when an extreme dark pattern is used, where the original dark pattern can be severely affected by the background illumination.

Fortunately, the proposed method builds a quantity lnE that responses to an ideal sinusoidal pattern in a much more linear way. As shown in Figure 5c, the optimally fitted line almost lies on top to the real responding curve. Consequently, an alleviated quoted error is expected (see Figure 5c) when we use this quantity in Equation (2) to measure the phase. The advantages of introducing the new quantity lnE in fringe measurement is further confirmed by Figure 5d, where lnE reduces the maximum, median, and even average quoted errors. As a result, an improvement in measurement precision is expected.

### 3.3. Determination of Optimal Responding Interval

A direct consequence of the weight function $w\left(G_{ij}\right)$ used in Equations (10)–(12) is the relatively large quote errors in the measurements of using very bright or dark patterns, as the fitting errors beyond the extreme pixel values $G_{min}$ or $G_{max}$ do not affect the minimization of the objective function O. Consequently, a larger deviation from the optimal linear response $g^{o}$ is expected when the camera responds to control values *Y* close to the extreme value −1 or 1. Its effect on the measurement system is quantitated by the maximum quote error $\mu_{max}$.

A possible and easy solution to alleviate this error would be by using only a smaller range of the responding curve, i.e., increasing $G_{min}$ and decreasing $G_{max}$. In this case, the fringe pattern, i.e., the periodic change from bright to dark, is quantitated by discrete integer (pixel) values from $G_{min}$ to $G_{max}$. Recall the principle of fringe pattern measurement, i.e., the periodicity in space with wavelength λ is represented by the changes of brightness which can be captured using imaging devices; a reduced measurement range [$G_{min}$, $G_{max}$] will degrade a system’s resolution ratio, which is defined as follows:

(14) $$k=\frac{\lambda }{G_{max}-G_{min}}\times 100\%$$

Both the resolution ratio and the maximum quote error are characteristics of a measurement system. In practice, a high resolution and small quote error are expected. However, these two characteristics contradict each other. Expanding the measurement range to increase the resolution ratio is accompanied by an increase in quote error. Thus, a compromise has to be made for a practical system. To this end, we combine the requirements of high resolution and small quote error into a unified expression $U=\mu_{max}+\eta \cdot k$, where η is a free parameter that scales the resolution *k* into an absolute unit.

The introduction of *U* allows us to express the pursuit of a better measurement system as an optimization problem, i.e.,

(15) $$\begin{array}{cc} \; & \mathrm{minimize}\;U(G_{min},G_{max})\\ \; & \mathrm{subject}\;\mathrm{to}\;G_{min}\in \left[0,255\right],\;G_{max}\in \left[0,255\right]\\ \; & \;G_{min}<G_{max} \end{array}$$

However, there is no easy way to find out the optimal measurement range that minimizes *U*. Fortunately, both $G_{min}$ and $G_{max}$ have a very limited range, which enables a complete search in finding the optimal solution. To proceed, for each pair of ($G_{min}$, $G_{max}{)}^{x}$, their corresponding control values ${\left(Y_{min},Y_{max}\right)}^{x}$ are first determined by utilizing the relation shown in Figure 2d. Instead of generating the periodic pattern with control values in the range of [−1.0, 1.0], we rescale the control function *Y* as

(16) $$Y=\frac{1}{2}Y_{max}\left(1+\cos\left(x\right)\right)+\frac{1}{2}Y_{min}\left(1-\cos\left(x\right)\right)$$

**Figure 6:**
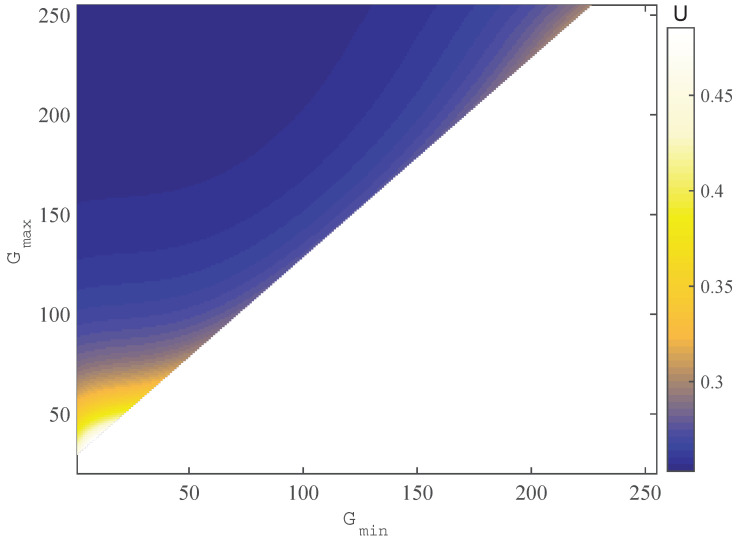
The distribution of function $U(G_{min},G_{max})$. The abscissa is the minimum gray value of the projection, and the ordinate is the maximum gray value of the projection.

A fixed colormap with limit values in the range [−1.0, 1.0] is then used to visualize the control value *Y*, which guarantees that the camera responds to the brightest and darkest pattern with pixel values $G_{min}$ and $G_{max}$, as long as the camera’s settings are unchanged. These preparing procedures allow a new solution for the optimization problem Equations (10)–(12) and, finally, the integral system error *U*. Scanning over all the possible pairs of $\left(Y_{min},Y_{max}\right)$, we come up with the distribution of integral errors *U* in the parameter space, which is illustrated in Figure 6. Recalling the requirement of a precise fringe pattern measurement system, we determine the optimal measurement range from 27 to 247 of the pixel values by finding out the minimum *U* in the parameter space.

## 4. Experimental Verification

The key advantage of the fringe reflection technique is its ability to provide a precise but easy-to-implement computer-vision-based relative position measurement. The periodic change in the brightness of the fringe pattern serves as a standard ruler in quantitating the position of a point in terms of wavelength λ or pattern size. Conventionally, the periodicity is captured by the pixel values *G* of the camera. The multiple nonlinear processes in the generation of the fringe pattern, as well as in image acquisition, make the pixel value representation not precise enough. The proposed method establishes a better linear representation of the fringe pattern. Thus, it is believed to give a more precise position measurement. This section presents the experimental results obtained from the implementation of the proposed method on a personal computer (Huawei MateBook 13, Manufactured in Shenzhen, China, Matab R2020a) to validate the effectiveness of the proposed method.

**Figure 7:**
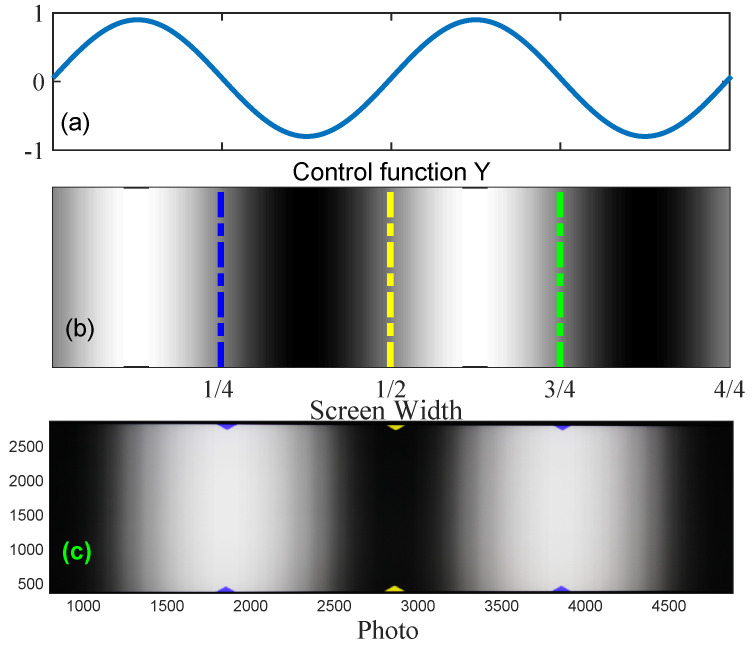
Illustrative description of validation experiment. (**a**) The control value of *Y* with optimized interval $Y_{min}$ and $Y_{max}$. (**b**) The generated fringe pattern displayed on a LCD monitor. The dashed lines mark the positions of 1/4, 1/2, and 3/4 of the screen lengths. (**c**) Fringe pattern captured using the camera. Pairs of ▽ − △ are used to mark the positions of the lines in panel (**b**).

To proceed, under the same illuminating condition, we generated a set of fringe patterns with a phase difference of π/2 using control value *Y* in the range [−0.893, −0.784] as determined previously (see Figure 7a). These patterns are displayed on an LCD screen (Dell D2720DS, Dell, manufactured in China) using full-screen mode. Recalling the purpose of the position representation of the fringe measurement, we validated the effectiveness of the proposed method by calculating the positions of a set of preset points of known positions (1/4, 1/2, and 3/4 of the screen length, marked by dashed lines in Figure 7b). To easily identify these positions, we marked the top and bottom terminals of the lines with colored pairs of ▽ − △. Thanks to the clear difference in colors, in comparison to the fringe patterns, these markers are easily identifiable in the photos acquired using the camera (Canon 60DES with iso 200, manufactured in Taiwan, China, see Figure 7c).

**Figure 8:**
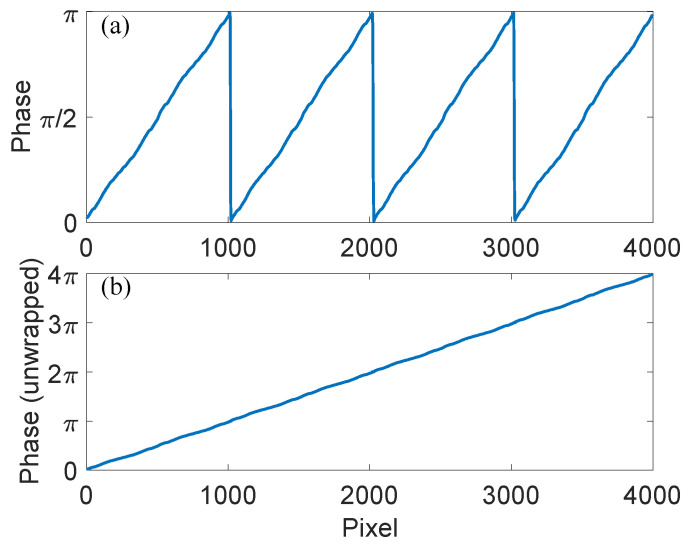
Phase measurement using the proposed method. (**a**) Wrapped phase at pixels along the horizontal direction extracted using the proposed quantity lnE, building upon the captured fringe patterns shown in Figure 7. (**b**) The unwrapped phase shows a very good linear increase.

We first demonstrate that the proposed quantity can help to extract the phase of each pixel point effectively. To this end, we calculated lnE using the expression obtained from Equations (10)–(12) and the pixel values of the fringe pattern captured by the camera. Instead of using $G_{i}$, we substituted the corresponding $\ln E_{i}$ in Equation (2). Figure 8 shows the obtained phase at each pixel point along the horizontal direction. Due to the definition of tan(·), the obtained phase wraps up π (see panel (a)). When we unwrap it, the obtained phase shows an almost linear increase with the horizontal length, i.e., number of pixels (see panel (b)).

**Figure 9:**
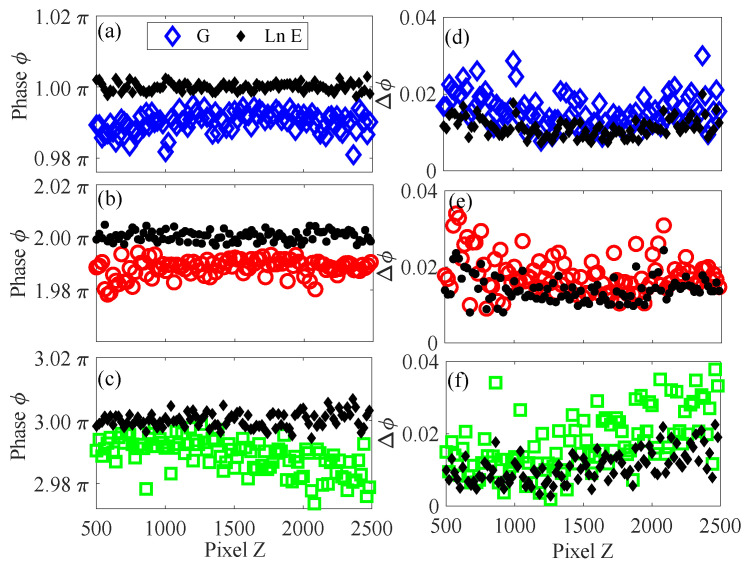
Panel (**a**)–(**c**). Phase measurements of the dashed lines in Figure 7 by using pixel value *G* (open symbols) and the scaled energy lnE (closed symbols). Panel (**d**)–(**f**). Measured phase errors of points along the dashed lines.

We then calculated the phase ϕ of each point on the lines between the tips of markers identified from the photos (see Figure 7c) using their pixel values *G* (open symbols) and scaled energy values lnE (closed small symbols). As these lines mark the positions of 1/4, 1/2, and 3/4 of the screen lengths, the absolute phase values of the points on the lines are known to be π, 2π, and 3π. Extracting the phase ϕ using the fringe patterns represented by the new quantity lnE provides a direct means of testing the effectiveness of the proposed method. Figure 9 shows the phase ϕ of the points along these lines calculated by using the pixel-based and lnE-based representation of fringe patterns. Comparing to the measurement results from pixel values (open symbols), the proposed method gives not only more precise results, but also smaller variations (see the black closed symbols). These superiorities of the proposed method are also confirmed by the smaller deviations of the extracted phases to the ground truth values, whose phases are π, 2π, and 3π, respectively. As there are multiple fringes (two wavelengths), the phases extracted by substituting pixel values as well as the scaled energy quantity lnE in Equation (1) are first unwrapped, and their differences to the ground truth values are calculated and plotted in the right panels of Figure 9. As shown in these figures, the proposed method (using scaled energy presentation) can significantly reduce the phase errors, with an average error of 0.011 radian, which is almost one-third smaller than the error of using pixel-value-based measurements.

**Figure 10:**
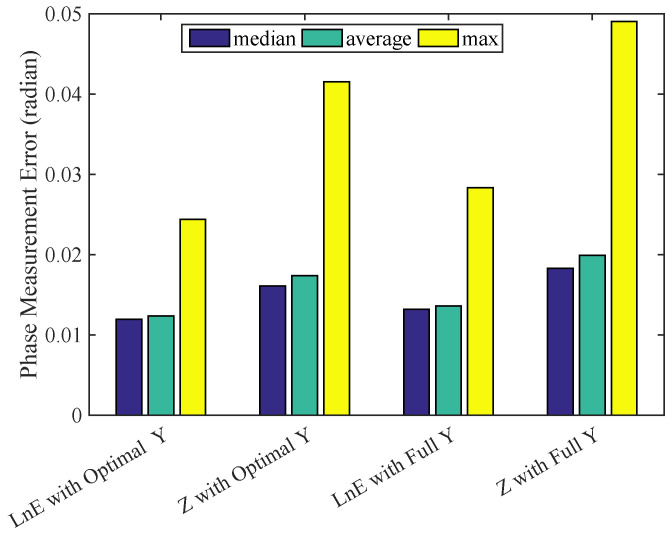
A comparison showing the effectiveness of the optimal control interval *Y* and the scaled energy representation in fringe pattern measurement.

We also test the effectiveness of the optimal control value interval by carrying out the same experiment presented above, expect that the fringe patterns are generated by using the control function Y=cos(·) of the range [−1.0 1.0], i.e., the full range of control value *Y*. We extract the median, average, and max of the measured phase errors calculated from the pixel values and the scaled energy lnE. A direct comparison is shown in Figure 10. Clearly, the optimized control interval of *Y* reduces measurement errors in both cases of using pixel values and scaled energy lnE. Most importantly, the combination of optimal control value *Y* in generating the fringe pattern and the scaled energy lnE representation of fringes, i.e., the proposed method, brings us the smallest measurement error.

## 5. Conclusions and Discussion

The fringe pattern is a key technology upon which many non-contact and precise measurement methods have been built. It is applicable in many different fields. The essential idea of fringe-pattern-based measurements is that the positional information can be extracted from the periodicity of the fringe patterns and their changes in phase. To obtain this information, photos of the fringe patterns are taken using digital cameras and the pixel values are typically used to represent the periodical changes in brightness. Although the phase-shift enables the elimination of the effect of background illumination and the easy extraction of positional information, the nonlinearity of the camera in responding to illumination cripples the measurement precision.

Targeting improved measurement precision, we first clarified the objective, i.e., the control value used to generate the fringe patterns, to which the camera should respond linearly. After analyzing the different physical processes involved in fringe pattern measurement, we came up with a scaled illuminating energy lnE to present the fringe pattern. By introducing a weight function ω, constructed from the difference of an ideal fitting between lnE and the control value *Y* in the extraction of a camera’s response function, we managed to construct the lnE from the measured pixel values. After determining the optimal measurement range, improvements in measurement precision are observed in comparison with the conventional pixel-value-based measurements.

Although we have not tested the proposed method in real applications, for example, in determining the integral quality of large reflective mirrors used in solar thermal power applications [14,18,37], the advantages of the proposed method are still obvious. To quantify the optical quality of mirrors effectively, the normal vectors of points on the reflective surface need to be determined through the pairing-up of the source points on the pattern-displaying screen and their reflections captured by the camera. Precisely representing each of the source point positions in the local coordinate system is the basis of the following procedures. The proposed method focuses on building a new quantity to better characterize the fundamental properties of the fringe pattern, by extracting the camera’s response curve to illumination. Although it was implemented on the sinusoidal pattern, it could be applicable to any other types of fringe pattern. The proposed method has shown its ability to provide more precise position measurements. Furthermore, the fringe technique only uses brightness in determining the position. Since the brightness is a continuous physical quantity, there is no limit on the number of points on the mirror surface that can be used. Ideally, the fringe pattern technique would allow the determination of all the normal vectors of points on a reflective mirror surface. This property brings a superior advantage over the color-coded technique [18], where the number of points on the surface that can be included in the quality examination is limited by the number of colors and the coding–decoding strategy.

The limitations of the proposed method mainly stem from the preparing procedures, which include extracting the camera’s response function and determining the optimal measurement range. As the camera’s response function is robust to operating parameters, i.e, exposure time, ISO values, etc., ([38]) the measurement system only needs to be calibrated once, as long as the background illumination condition remains unchanged. As a result, the application of the proposed method requires a relatively stable illumination condition, i.e., the measurement might be vulnerable to external light disturbances. Fortunately, this condition can be easily guaranteed, even when this method is applied to assess the quality of a large reflective mirror used in solar thermal power, as production lines are typically indoors.

## Data Availability

Data are contained within the article.
